# MiR-148b suppresses cell proliferation and invasion in hepatocellular carcinoma by targeting WNT1/β-catenin pathway

**DOI:** 10.1038/srep08087

**Published:** 2015-01-28

**Authors:** Jun-gang Zhang, Ying Shi, De-fei Hong, Mengqi Song, Dongsheng Huang, Chun-you Wang, Gang Zhao

**Affiliations:** 1 Pancreatic Disease Institute, Union Hospital, Tongji Medical College, Huazhong University of Science and Technology, Wuhan, 430022, China; 2 Hepatobiliary and Pancreatic Surgery, Zhejiang Provincial People's Hospital, Hangzhou, 310014, China; 3 Obstetrics and Gynecology, Zhejiang Provincial People's Hospital, Hangzhou, 310014, China; 4 Hepatobiliary Surgery, Union Hospital, Tongji Medical College, Huazhong University of Science and Technology, Wuhan, 430022, China

## Abstract

Accumulating evidences indicate that microRNAs play a vital role in regulating tumor progression. However, the roles of miR-148b in hepatocellular carcinoma (HCC) are still largely unknown. In this study, our data showed that miR-148b was significantly downregulated in 40 pairs of human HCC tissues. Further, the deregulated miR-148b was significantly correlated with larger tumor size, more tumor number, metastasis and worse prognosis in HCC. Overexpression of miR-148b inhibited HCC HepG2 cells proliferation and tumorigenicity. Further, miR-148b induced cells apoptosis by activating caspase- 3 and caspase-9, and induced S phase arrest by regulating cyclinD1 and p21, and also inhibited cell invasion. Data from the dual-luciferase reporter gene assay showed that WNT1 was a direct target of miR-148b, and overexpressed WNT1 inversely correlated with miR-148b levels in HCC tissues. Silencing of WNT1 inhibited the growth of HCC cells, and also induced cells apoptosis and inhibited invasion, which is consistent with the effects of miR-148b overexpression. MiR-148b downregulated expression of WNT1, β-catenin and C-myc, while upregulated E-cadherin expression. We conclude that the frequently downregulated miR-148b can regulate WNT1/β-catenin signalling pathway and function as a tumor suppressor in HCC. These findings suggest that miR-148b may serve as a novel therapeutic target for HCC.

Hepatocellular carcinoma (HCC) is the third leading cause of cancer deaths and has an increasing incidence worldwide^1^,^2^. The high incidence of HCC is particularly in Chinese population due to endemic Hepatitis B virus infection^3^. Liver transplantation and liver resection are the curative treatments for early-stage HCC. HCC carcinogenesis is a multistep process through accumulation of genetic and epigenetic alterations. Although the risk factors for HCC are well characterized, the molecular pathogenesis is not well understood^4^,^5^. Thus, the identification of new possible targets for preventing the initiation and progression of HCC is imperative and must be improved.

MicroRNAs (miRNAs) are small non-coding RNAs that regulate target gene expression by binding to the 3′-untranslated region (3′-UTR) of their target mRNAs, resulting in either mRNA degradation or translational repression^6^,^7^. MiRNAs play essential roles in many normal biological processes, including cell proliferation, apoptosis, differentiation and stress resistance^8^,^9^. Accumulating evidence has shown that dysregulation of miRNAs expression is associated with oncogenic transformation. MiRNAs have been identified in many types of tumors and function as oncogenes or tumor suppressors involved in cancer development and progression^10^,^11^,^12^, and miRNAs are emerging as targets for cancer molecular therapy.

Microarray studies have identified a number of miRNAs that are dysregulated in HCC. Gramantieri L et al and other groups discovered several miRNAs, which exhibited significant, differential expression pattern in HCC^13^,^14^,^15^. Recently, miR-148b has been found to be dysregulated in some types of cancers, such as downregulated in colorectal cancer^16^, gastric cancer^17^, breast cancer^18^ and pancreatic cancer^19^,but overexpressed in ovarian cancer^20^. Zhang Z et al showed that miR-148b expression was significantly decreased in HCC tissues and was associated with poor overall survival of patients^21^. However, the exact function of miR-148b in hepatocarcinogenesis has not been revealed yet.

WNT1, has been shown to regulate the progression of cancer because it promotes cell proliferation, migration and prolongs cancer cell survival^22^,^23^. WNT1 binds to specific Frizzled (FZD) surface receptors of target cells to activate distinct intracellular pathways, resulting in the accumulation and nuclear localization of down-stream molecule β-catenin protein. Nuclear β-catenin induces the expression of target genes such as E-cadherin and c-Myc^24^,^25^, which have been characterized to be critical for cancer development^26^,^27^. Wnt antagonist remarkable inhibited cancer cells invasion and induced the expression of Wnt/β-catenin transcriptional target gene E-cadherin^28^. Some clinical studies have reported that abnormal activation of Wnt/β-catenin pathway is frequently involved in hepatocarcinogenesis^29^,^30^. Therefore, we selected the WNT1 as the potential target for miR-148b to further explore the effects of miR-148b/Wnt/β-catenin signal on HCC.

## Results

### Clinicopathologic Significance of miR-148b in HCC Patients

**Figure 1:**
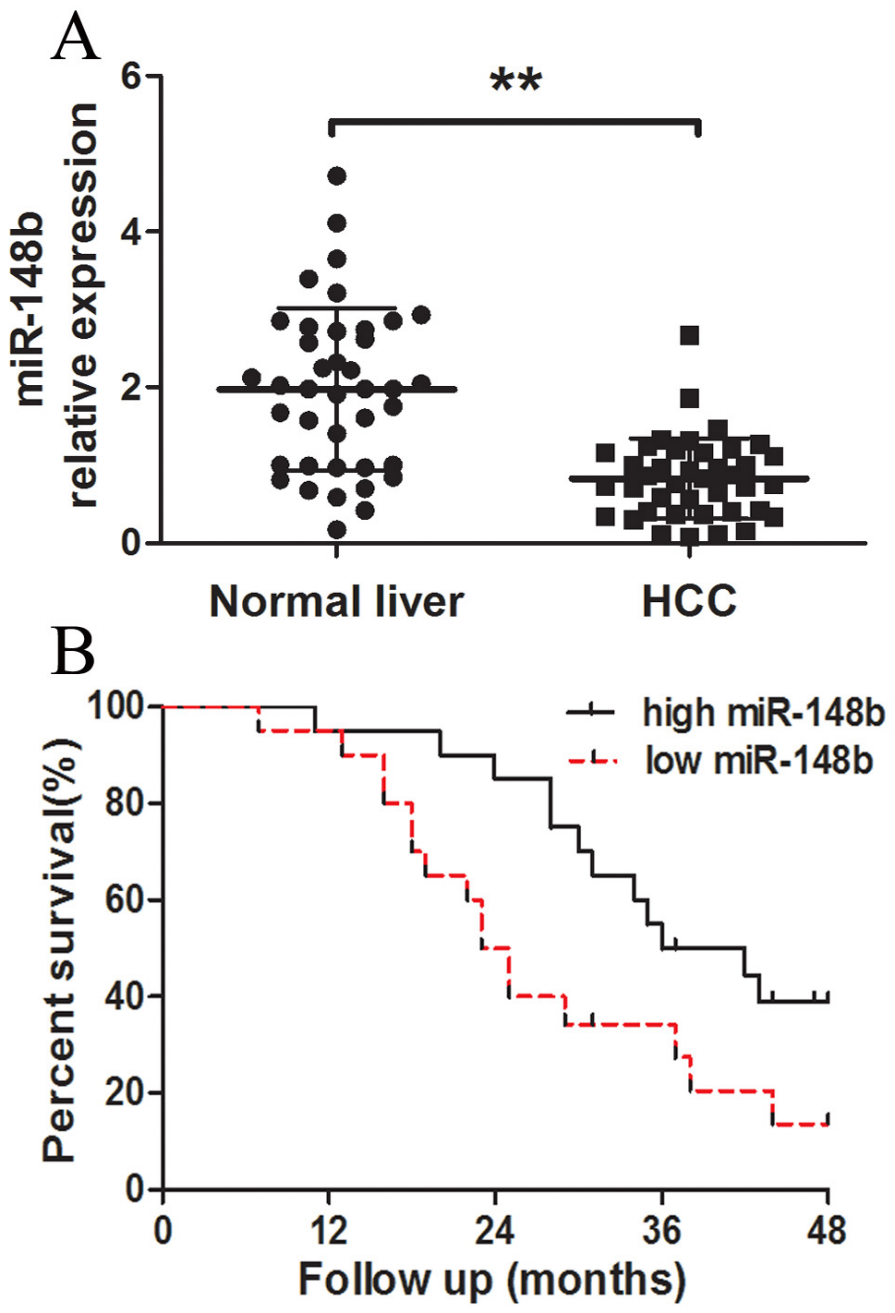
Analysis of miR-148b expression in human HCC tissues and survivals of HCC patients. (A) The relative expression levels of miR-148b in human HCC tissues (n = 40) and matched normal liver tissues (n = 40) were detected by qRT-PCR. U6 was used as the control for RNA loading, and miR-148b abundance was normalized to U6 RNA. (B) Kaplan- Meier curves of overall survivals of 40 HCC patients, according to miR-148b expression scored as low expression (below the median value, n = 20) and high expression level (above the median value, n = 20). MiR-148b downregulation correlated significantly with shorter overall survivals. P-value was shown with log-rank test. **P<0.01.

MiR-148b was detected in 40 cases of HCC tissues and noncancerous liver tissues using a qRT-PCR method. As shown in Fig. 1A, the expression level of miR-148b in HCC tissues was significantly decreased in comparison with the expression level in noncancerous tissues (P < 0.05). The Kapan-Meier survival analysis revealed that the patients with low miR-148b expression had a significantly poorer prognosis than those with high miR-148b expression (Fig. 1B, P<0.05). We further studied the correlation between miR-148b and clinical pathological characteristics of HCC. The low miR-148b expression group showed a higher incidence of increased tumor size (P = 0.003), tumor number (P = 0.011) and metastasis (P < 0.001). However, no significant differences were observed with respect to sex, age, AFP, histologic grade, TNM stage or HBV in HCC (Table 1). All these results indicate the importance of miR-148b downregulation in HCC progression.

### Over-expression of miR-148b suppresses proliferation of HepG2 cells in vitro and in vivo

**Figure 2:**
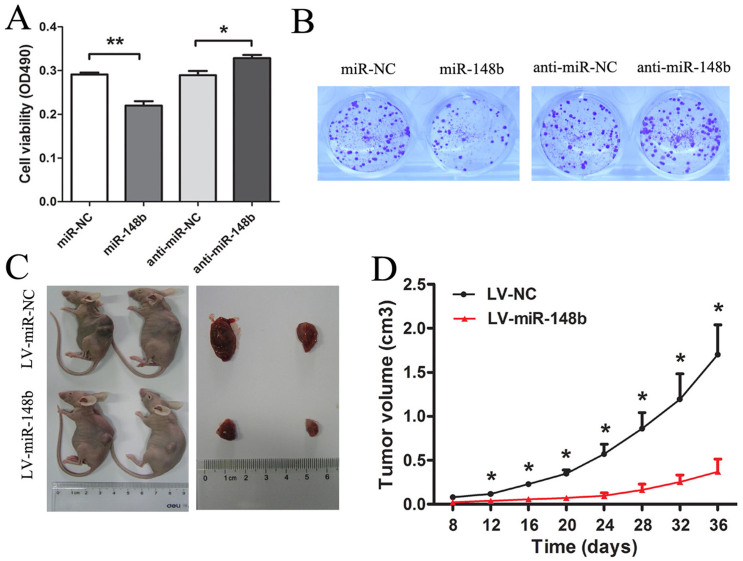
MiR-148b inhibited proliferation of HCC cells in vitro and tumorigenicity in vivo. (A) The effect of transient transfection of miR-148b mimics (miR-148b) or miR-138 inhibitor (anti-miR-148b) and their respective negative control (miR-NC or anti-miR-NC) (50 nM) for 48 h on the growth of HepG2 cells was examined by MTT assay. (B) The effect of miR-148b on proliferation of HepG2 cell was measured by colony formation assay. The colonies were evaluated according to the ratio between lentiviral miR-148b (LV-miR-148b)-infected cells and empty lentiviral vector (LV-NC)–infected cells at a multiplicity of infection of 10. Each data is representative of three independent experiments. The LV-miR-148b or LV-NC transfected HepG2 cells were injected subcutaneously into nude mice, respectively. Excised tumor volumes (C) at 36 days after implantation and tumor growth curves (D) were shown as indicated. Data were shown as the Mean ± SD of 10 mice. *P<0.05; **P<0.01.

We evaluated the functional role of miR-148b in HCC cells by measuring cell proliferation in HepG2 cells. MiR-148b was significantly up-regulated following transfection with miR-148b mimics and down-regulated by transfection with miR-148b inhibitor (Fig. S1). MTT assays were employed to detect the proliferation of HepG2 cells. Ectopic expression of miR-148b inhibited cell proliferation while downregulation of miR-148b promoted cell proliferation at 48 h (Fig. 2A). We also observed that transfection with LV-miR-148b led to a significant reduction of colony counts of HepG2 cells than transfection with LV-miR-NC (*P* < 0.01) (Fig. 2B). Furthermore, the inhibitory effect on the growth of HCC cells was further confirmed by tumorigenicity *in vivo*. After 36 days, the tumor volume of nude mice subcutaneously injected with LV-NC transfected cells was 623.97 ± 25.05 mm^3^, whereas the tumor size of mice injected with LV-miR-148b cells was 148.89 ± 28.46 mm^3^ (P < 0.05; Fig. 2C). Throughout the tumorigenic period, tumors from the LV-miR-148b transfected cells grew significantly slower (Fig. 2D). QRT-PCR results demonstrated that there was an obviously increase of miR-148b expression in LV-miR-148b transfected tumors compared with control tumors (Fig. S2). These results infer that miR-148b functions as a potential tumor suppressor in HCC carcinogenesis.

### MiR-148b Induces Apoptosis and Cell Cycle Arrest and Inhibits Invasion in HCC Cells

**Figure 3:**
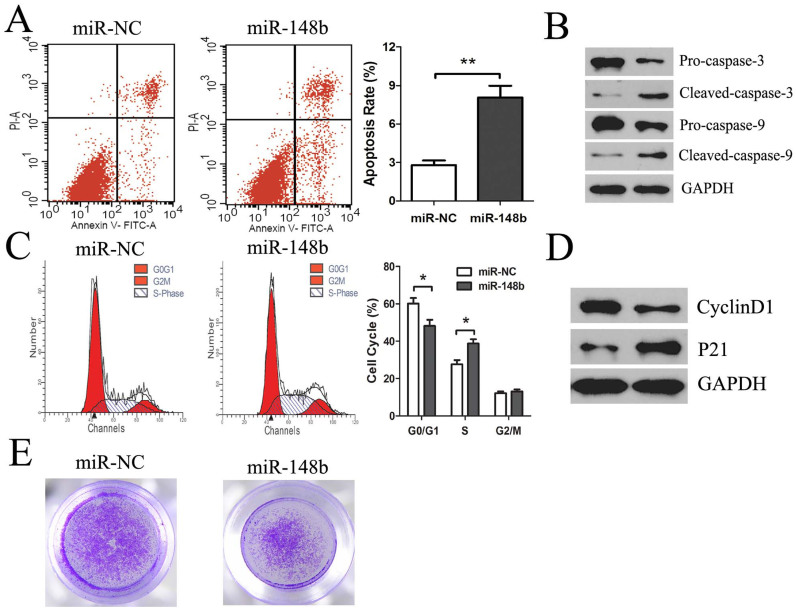
The effects of miR-148b on in vitro apoptosis, cell cycle arrest and invasion of HCC cells. After transfection with miR-148b or miR-NC (50 nM) for 48 h, (A) the apoptosis of HepG2 cells was measured by Annexin V staining and flow cytometry (the right lower quadrant represents apoptosis), and (B) representative western blotting images for detecting the protein levels of Pro-caspase-3 and Pro-caspase-9, cleaved caspase-3 and caspase-9. GAPDH served as the loading control. HepG2 cells were treated as above, cell cycle distribution was measured by PI staining and flow cytometry (C) and cell cycle protein cyclinD1 and p21expression (D). (E) HepG2 cells were treated as above, and the cell invasion ability was measured by matrigel invasion assays. Data were representative of three independent experiments. *P<0.05; **P<0.01.

To investigate the potential mechanism responsible for the observed effects of miR-148b on the proliferation of HCC cells, we evaluated apoptosis rate in HepG2 cells transfected with miR-148b or miR-NC. Compared to miR-NC, miR-148b transfection resulted in a significant increase in apoptosis rate (8.07 ± 0.92 vs 2.79 ± 0.36, *P* < 0.01) (Fig. 3A). To further determine the mechanisms of miR-148b-induced apoptosis, we detected the expression of apoptosis-related caspase family protein. MiR-148b consistently induced a significant reduction of Pro-caspase-3 and Pro-caspase-9 and increased the cleavage of caspase-3 and caspase-9, which suggests that miR-148b induces apoptosis of HCC cells through activating the caspase- 3 and caspase-9 (Fig. 3B).

Moreover, there was a significant increase in the S phase (38.75 ± 2.26 vs 27.66 ± 2.17, *P* < 0.01) and decrease in the G0/G1 phase (Fig. 3C). As shown in Fig. 3D, miR-148b decreased cyclinD1 and upregulated p21 protein expression, which participate in cell cycle regulation. These results indicate miR-148b could induce cell cycle arrest at S phase by regulating cyclinD1 and p21.

To study the role of miR-148b in inhibiting invasive ability, we transfected HepG2 cells with miR-148b mimics and performed matrigel invasion assays. We observed that HepG2 cells invasive ability was significantly inhibited following transfection with miR-148b (Fig. 3E). These results revealed that miR-148b mediated invasion of HCC cells.

### WNT1 is a direct target of miR-148b

**Figure 4:**
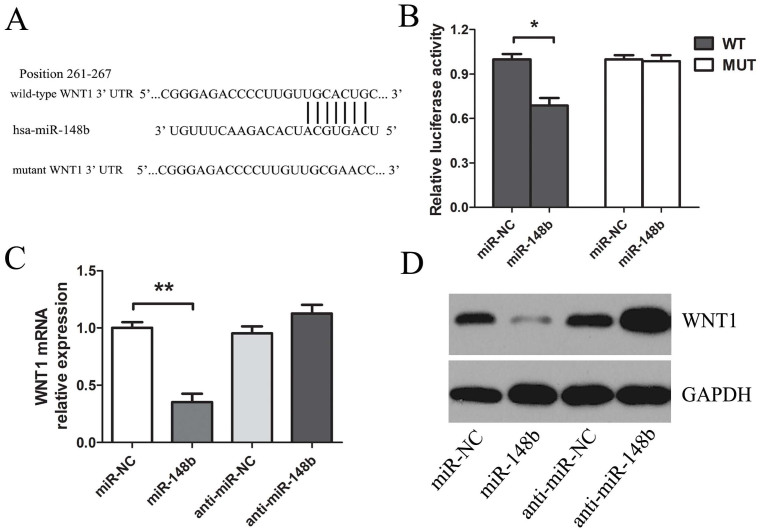
WNT1 was the direct target of miR-148b in HCC cells. (A. Top) Human WNT1 3′UTR fragment containing wild-type or mutated miR-148b–binding sequence. (WT: wild type; MUT: mutant type). (A. Bottom) MiR-148b and its putative binding site in the 3′UTR of WNT1. (B) Luciferase reporter assays, with co-transfection of wild-type or mutant 3′UTR (100 ng) and miR-148b or miR-NC (50 nM) in HepG2 cells as indicated. Firefly luciferase activity was normalized by Renilla luciferase activity. After transfection with miR-148b or anti-miR-148b (50 nM) for 48 h, qRT-PCR (C) and western blot (D) were used for monitoring WNT1 expression in HepG2 cells. *P<0.05; **P<0.01.

To fully understand the mechanisms by which miR-148b executed its function, we adopted the bioinformatic algorithms (MiRanda, TargetScan, and PicTar) for target gene prediction. Among these candidates, WNT1, an important oncogene, was identified as one of the potential targets of miR-148b and selected for further analysis. The predicted binding site of miR-148b was observed in the 3′-UTR of WNT1 mRNA (Fig. 4A). To test that WNT1may be a direct target of miR-148b, the reporter plasmid harboring the wild-type or mutant 3′-UTR region of WNT1 was constructed. Our results showed that miR-148b significantly decreased the firefly luciferase activity of the reporter with wild type 3′UTR, but unaffected the activity of mutant type 3′UTR vector, which suggests that the 3′UTR region of WNT1 was fully responsible for miR-148b function (Fig. 4B). To further investigate the interaction between miR-148b and WNT1, qRT-PCR and western blotting analysis was conducted to measure the effect of miR-148b on endogenous WNT1 expression. We found that WNT1 was downregulated in miR-148b-treated HepG2 cells in mRNA and protein levels (Fig. 4C). These data suggest that miR-148b directly perfectly recognizes the 3′-UTR of WNT1 mRNA and interferes with mRNA degradation and translation in HCC cells. Therefore, downregulated miR-148b in HCC inhibits the suppression of WNT1, which in turn accelerates tumorigenesis.

### WNT1 inversely correlates with miR-148b levels

**Figure 5:**
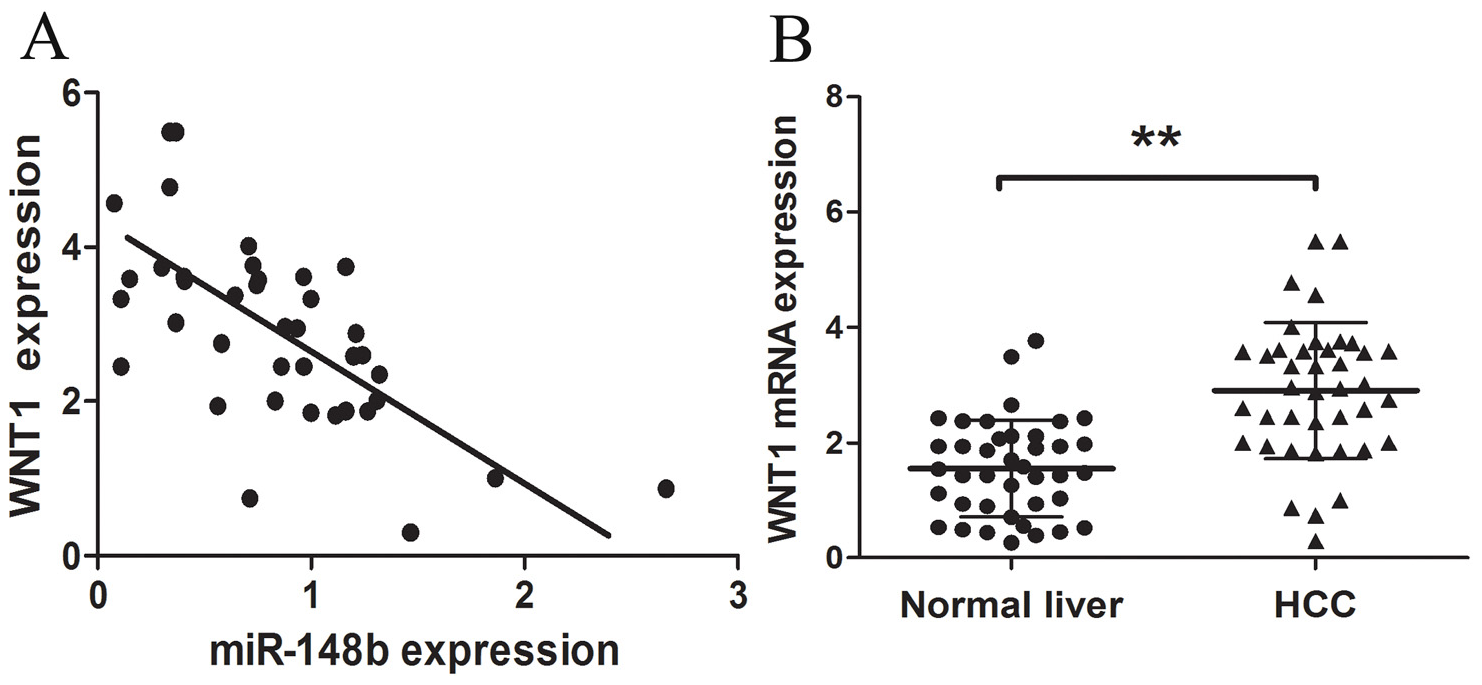
WNT1 inversely correlated with miR-148b levels in HCC tissues. (A) WNT1 was detected in 40 HCC tissues and matched normal liver tissues by qRT-PCR, and the WNT1 abundance was normalized to GAPDH. (B) The relationship between WNT1 and miR-148b expression was explored by Spearman's correlation analysis in HCC tissues. *P<0.05; **P<0.01.

Previous results have shown that miR-148b can directly inhibit WNT1 expression. To further validate whether the deregulated miR-148b can increase WNT1 levels and facilitate HCC progression, we assessed the WNT1expression and the correlation between WNT1 and miR-148b expression in HCC. The result of qRT-PCR showed that the average expression of WNT1 was significantly higher in HCC tissues than in normal liver tissues (P<0.01, Fig. 5A). As shown in Fig. 5B, a significant inverse correlation was observed between WNT1 and miR-148b expression in 40 HCC tissues by Spearman's correlation (r = −0.6352; P < 0.001). These results further confirmed that endogenous WNT1 was regulated by miR-148b.

### MiR-148b targeted WNT1 contributes to growth and invasion of HCC cells

**Figure 6:**
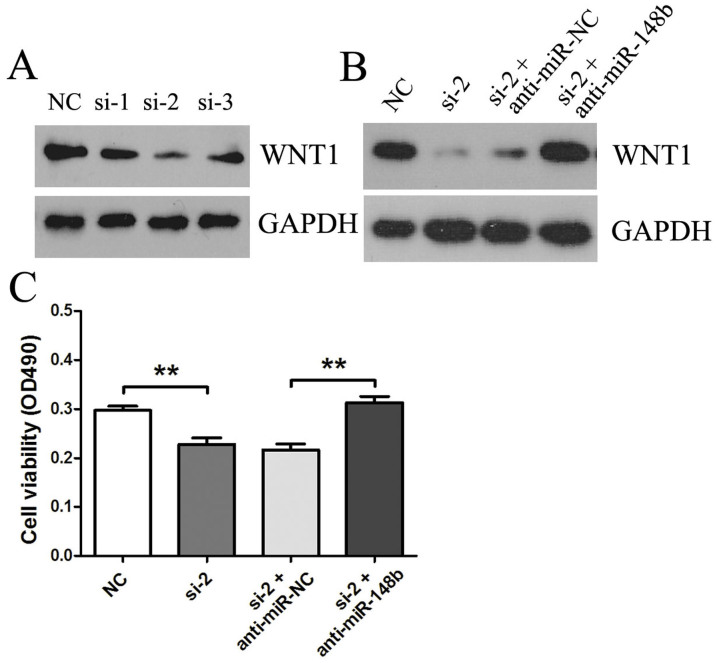
MiR-148b targeted WNT1 contributed to HCC cells growth. (A) The WNT1expression was examined by western blotting in HepG2 cells transfected with 3 different siRNAs targeting WNT1 (si-WNT1-1, si-WNT1-2 and si-WNT1-3) or NC. (B) The effect of transfection of si-WNT1 and anti-miR-148b targeting WNT1 in HepG2 cells was examined by western blotting at 48 h following transfection. (C) The effect of anti-miR-148b on si-WNT1-induced proliferation inhibition of HepG2 cells was examined by MTT assays 48 h following transfection. All results were reproducible in three independent experiments. *P<0.05, **P<0.01.

To explore whether miR-148b regulated growth of HCC cells through deregulating WNT1, we studied the effects of silencing WNT1 on the growth of HepG2 cells. The result of western blotting demonstrated that all of 3 siRNAs targeting WNT1 were able to effectively knockdown WNT1 expression (Fig. 6A) and anti-miR-148b effectively rescued WNT1-siRNA silencing of WNT1in protein levels (Fig. 6B). The MTT assays showed that the silencing of WNT1 significantly reduced growth of HepG2 cells relative to the control. Meanwhile, the proliferation inhibition of WNT1 siRNA could be rescued by anti-miR-148b (Fig. 6C). To further understand the mechanism by which cells proliferation is affected by WNT1, flow cytometry was performed to analyze the apoptosis. Compared to NC group, WNT1-RNAi increased HepG2 cell apoptosis (Fig. S3A). We also observed that HepG2 cells invasive ability was significantly inhibited following WNT1-RNAi (Fig. S3B). These results were consistent with the effect of miR-148b overexpression, indicating that miR-148b regulates HCC cells growth, apoptosis and invasion by the direct target of WNT1.

### MiR-148b modifies the downstream signaling pathway of WNT1

**Figure 7:**
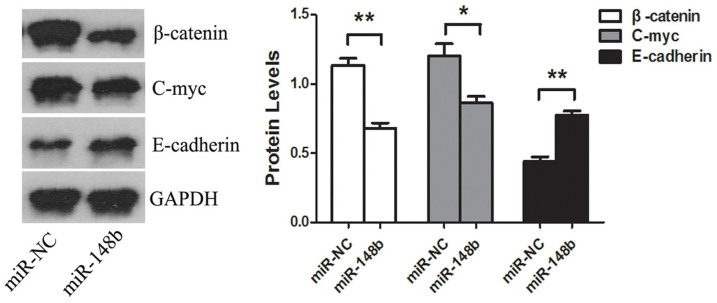
The effects of miR-148b on the downstream signaling pathway of WNT1. HepG2 cells were transfected with miR-148b or miR-NC for 48 h, the expression levels of β-catenin, C-myc and E-cadherin proteins in HepG2 cells were detected by western blotting. The results were standardized against the levels of GAPDH and repeated three times, *P < 0.05, **P < 0.01.

To investigate the mechanism responsible for the observed effects of miR-148b, we further tested the downstream signaling pathway of WNT1. We demonstrated that transfection with miR-148b had significantly downregulated β-catenin, C-myc and upregulated E-cadherin protein levels, compared with miR-NC (P < 0.05, Fig. 7).

## Discussion

MiRNA profiles have been established for a variety of solid and hematologic malignancies^31^,^32^. The frequent aberrant expression of miRNAs implies that they have a tumor suppressor or oncogene function. Recently, it has been noted that the expression profiles of miRNAs can be used for diagnosis and prognosis of human malignancies^33^,^34^. Song Y et al found that miR-148b is downregulated and suppresses colorectal cancer cell proliferation and tumorigenicity by targeting the cholecystokinin-2 receptor gene (CCK2R)^16^. Our previous reports suggested that miR-148b is also downregulated in pancreatic cancer, and it suppresses pancreatic cancer development through directly targeting AMPKα1^35^. However, Cuk K et al found that miR-148b is significantly upregulated in the plasma of breast cancer patients and may be used for the efficient diagnosis^36^. Previously, miR-148b is suggested to be upregulated in ovarian cancer, and its upregulation may be involved in the early stage of ovarian carcinogenesis^20^. These controversial results of miR-148b in cancer development may reflect the diverse roles of miR-148b in different types of cancer. In the present study, we identified that miR-148b is a predominantly downregulated miRNA in HCC tissues compared with normal liver tissues by using qRT-PCR assay, suggesting that miR-148b might be implicated in tumorigenesis.

Downregulation of miR-148b is further suggested to correlate with shorter disease-free survival of HCC patients. Hence, HCC patients with lower miR-148b expression in tumor tissues are shown to have a worse prognosis. Meanwhile, our clinical data showed that miR-148b deregulation was significantly associated with larger tumor size, more tumor number and metastasis. Thus, determination of miR-148b expression levels in HCC tissues may be a novel biomarker to predict and identify the prognosis and progression of HCC patients.

As is commonly known, uncontrolled cell proliferation is the key mechanism for neoplastic progression^37^. Recently, restoration of miRNAs is believed to have substantial clinical potential in cancer therapy^38^. Our results from both MTT assays indicated that miR-148b significantly inhibited the growth of HCC cells in vitro. This was further supported by the result that overexpression of miR-148b could inhibit the growth of xenograft tumors in vivo. The ability to evade apoptosis is always a hallmark of cancer cells. MiRNAs prevent tumor development usually by negatively regulating oncogenes that control apoptosis or cell cycle. As we known, mitochondrial pathway (the mainly apoptosis pathway), is characterized by the efflux of cytochrome C from the mitochondria to the cytosol, where the initiator caspase-9 is activated and triggers the activation of caspase-3^39^. Our data showed that miR-148b induced HepG2 cell apoptosis through activating the caspase- 3 and caspase-9. Furthermore, we found that miR-148b induced cell cycle arresting at S phase by regulating cyclinD1 and p21, which play the important role in cell cycle regulation. Invasion and metastasis are the major causes of miserable prognosis in patients with HCC. Our data showed that restoration of miR-148b inhibited invasion of HCC cells. These data suggest that miR-148b functioned as a tumor suppressor in HCC cells and involved in HCC progress.

To explore the role and mechanism of miR-148b in HCC, we next set out to identify the putative target genes of miR-148b. WNT1, the first member of the 19 known human Wnt family, has been abnormally expressed in a wide variety of human tumors including HCC^40^,^41^,^42^. According to bioinformatics analysis, we identified WNT1, as miR-148b putative target gene. In the present study, an important molecular association between miR-148b and WNT1 was demonstrated. (A) Overexpression of miR-148b decreased the luciferase reporter activity of wild-type 3′UTR but not mutant 3′UTR of WNT1. (B) Up-regulation of miR-148b significantly reduced WNT1 mRNA and protein levels in HCC cells, but down-regulation of miR-148b increased WNT1 levels. (C) The mRNA levels of WNT1 inversely correlated with miR-148b levels in 40 HCC tissues. (D) Silencing of WNT1 reduced growth, induced apoptosis and inhibited invasion of HCC cells, which was similar to the phenotypes induced by miR-148b overexpression. Furthermore, the proliferation inhibition of WNT1 siRNA could be rescued by anti-miR-148b. (E) The downregulation of WNT1 expression by miR-148b inhibited the constitutive expression of β-catenin, C-myc and enforced E-cadherin levels, downstream molecules in WNT signaling. Taken together, these data demonstrate that miR-148b can regulate WNT1 expression and function as a tumor suppressor in HCC development.

In conclusion, our present study demonstrates that the deregulated expression of miR-148b is associated with poor prognosis and aggressive phenotype of HCC. The effects of miR-148b on HCC cell proliferation and invasion observed in this study may be partially due to its regulation of WNT1/β-catenin signalling pathway. These findings may provide the molecular mechanism of miR-148b in tumorigenesis and invasion/metastasis of HCC. All of these results indicate miR-148b may be applied as potential prognostic biomarker and new therapeutic target in HCC.

## Methods

### Clinical specimens

Surgically resected HCC tissues and matched adjacent normal liver tissues were collected from 40 primary HCC patients during operation in 2009 at Hepatobiliary Surgery, Union Hospital (Wuhan, China). None of the patients had received chemotherapy or radiotherapy prior to liver resection. All specimens were immediately frozen in liquid nitrogen and then stored at −80°C until analysis.

The complete details of the entire study design and procedures involved were in accordance with the Declaration of Helsinki. All participants and their parents gave their written informed consent to participate in the study after the risks and benefits we discussed in detail. This study was approved by the ethics committee of the Union Hospital of Huazhong University of Science and Technology.

### Cell lines and cell culture

Human HCC cell line HepG2 was obtained from American Type Culture Collection (ATCC). Cells were maintained in RPMI-1640 mediums supplemented with 10% fetal bovine serum (FBS) in a humidified incubator at 37°C containing 5% CO_2_.

### Cell viability assay

Cell viability of HCC HepG2 cell was measured using the MTT method as previously described^43^. In brief, cells were seeded in 96-well plates at densities of 5 × 10^3^ cells per well and treated with 50nM miR-148b for 48 h. At the end of transfection, cells were treated by using MTT dye (5 mg/mL) for 4 h, and then the medium was replaced by 150 uL DMSO. The absorbance was measured using an activation wavelength of 570 nm. Cell viability was determined by comparison with control cells for three independent experiments, each of which used *n* = 5 replicate wells per assay condition.

### Colony formation assay

300 transfected pancreatic cancer cells were grown in a 12-well plate and allowed to grow for 12 days in RPMI-1640 containing 10% FBS. Colonies were fixed with methanol and then stained with 0.1% crystal violet solution, and the number of total colonies was counted under a microscope. Colonies of more than 50 cells were counted.

### Evaluation of apoptosis and cell cycle distribution

Apoptosis and cell cycle distribution were determined by FACS flow cytometry as previously described^35^. Briefly, for apoptosis analysis, cells were collected by 0.25% trypsinization, washed twice with cold phosphate buffered saline (PBS) and resuspended with binding buffer at cell density of 5 × 10^5^/ml. Cells were then stained with 5 ìL Annexin V-FITC and 5 ìL PI according to the manufacturer's protocol. The Samples were acquired by the FACS flow cytometer (BD Biosciences) and analyzed with Cellquest software. For cell cycle analysis, cells were harvested, washed twice by cold PBS and fixed in 70% cold ethanol overnight at −20°C. Then cells were treated with staining solution for DNA content (Propidium Iodide, 3.4 mM Tris-Cl (pH 7.4), 100 μg/ml RNase A and 0.1% triton X-100 buffer). Populations in G0/G1, S and G2/M phase were measured with FACS flow cytometry.

### In vitro invasion assay

Cell invasion was assessed using the Matrigel Invasion Chamber of pore size 8 mm (Corning, UK) as previously described^44^. In brief, a total of 5 × 10^4^ cells transfected in medium containing 0.1% bovine serum albumin were seeded into the upper chamber pre-coated with matrigel (Sigma). Medium containing 30% fetal bovine serum was placed in the lower chamber. After 48 hours, the invasive cells at the bottom of the membrane were fixed with 4% paraformaldehyde solution and then stained with 0.5% crystal violet, and cells that did not pass through the filter were removed with cotton swabs. Invasive cells on the lower surface of the membrane were photographed at a magnification of 400 × in five random fields and then counted manually for each treatment.

### Western blotting

Western *Blotting* was performed as described recently^45^ with some modifications. Whole cell extracts were prepared in a lysis buffer, and cellular protein was measured using a BCA protein assay kit (Pierce, Bonn, Germany) according to the manufacturer's instruction. Total cellular protein (40 μg) were separated by 10% SDS–polyacrylamide gels, and transferred onto nitrocellulose (Millipore, Billerica, MA, USA) membranes. After blocking in 5% skim milk for 1.5 h, the membranes were incubated with primary antibodies overnight at 4°C (anti-WNT1, anti-C-myc, anti-procaspase-3, anti-cleaved caspase-3, anti-procaspase-9, anti- cleaved caspase-9, anti-CyclinD1 and anti-P21,1:500; anti-*β*-catenin and anti-GAPDH,1:1000). After washing twice with Tris-buffered saline containing 0.1% Tween 20, the membrane was incubated with the HRP-conjugated secondary antibody for 2 h at room temperature and proteins bands were visualized on Kodak X-ray film using enhanced chemiluminescence ECL substrate (Pierce).

### Transfection

MiR-148b mimics (miR-148b), miR-148b negative control (miR-NC), miR-148b inhibitor (anti-miR-148b), miR-148b inhibitor negative control (anti-miR-NC) and siRNA duplexes targeting human WNT1 (si-WNT1-1, si-WNT1-2 and si-WNT1-3) were designed and synthesized by RiboBio (Ribobio Co., Guangzhou, China). SiRNA duplexes with non-specific sequences were used as siRNA negative control (NC). The transfections were carried out using Lipofectamine-RNAi MAX (Invitrogen) following the manufacturer's protocol. 5 × 10^4^ HepG2 cells in 12-well plate were transfected with indicated miRNA duplex (Ribobio Co.) and collected 48 h after transfection for assay. Lentiviral miR-148b (LV-miR-148b) and empty lentiviral vector (LV-NC) were constructed by Genechem Company (Shanghai, China), and transfected to HepG2 cells according to the manufacturer's instruction. Lentiviral transfection efficiency (>90%) was confirmed by fluorescent micros-copy. The siRNAs-targeted human WNT-1 is designed from the mRNA sequences of human WNT-1 gene (GenBank: NM_005430). Other oligonucleotide sequences are listed previously^35^.

### Dual-luciferase reporter assay

HepG2 cells (1 × 10^4^) seeded in 96-well plate were cotransfected with50 ng of wild type pGL3-WNT1-3′UTR or mutant pGL3- WNT1-3′UTR and 50 nmol/L miR-148b mimic or miR-NC by using Lipofectamine-2000 transfection reagent. Cells were collected 48 h after transfection, and luciferase activity was measured using a dual-luciferase reporter assay system (Promega, Madison, WI). Firefly luciferase activity was normalized to its corresponding Renilla luciferase activity. All experiments were performed in duplicate and repeated at least in three independent as described previously^35^.

### HCC xenograft model

LV-NC or LV-miR-148b transfected HepG2 cells (5 × 10^6^ cells) in 150 uL PBS were inoculated subcutaneously into the right flank of 4-week-old female BALB/c nude mice subcutaneously (n = 6 mice per group). Tumor growth was measured every 4 days from injection using a digital caliper and the tumor volume was calculated by the formula: tumor volume = (length × width^2^)/2^46^. Nude mice were sacrificed at the end of the treatment period, and xenografts harvested. All experimental procedures involving animals were performed in accordance with the Guide for the Care and Use of Laboratory Animals and were approved by Animal Care and Use Committee of Tongji Medical College of Huazhong University of Science and Technology.

### Quantification of miR-148b and WNT1 mRNA expression

Total RNA was extracted from cultured cells and tissues with Trizol reagent according to the manufacturer's instruction (Invitrogen, Carlsbad, CA). To quantify mature miR-148b expression, total RNA was polyadenylated using poly-A polymerase based First-Strand Synthesis kit (TaKaRa Bio, Japan) according to the manufacturer's protocol. To quantify WNT1 expression, total RNA (1ug) was converted to cDNA using M-MLV reverse transcriptase (Invitrogen). After reverse transcription, qRT-PCR was performed using the quantitative SYBR Green PCR kit (TaKaRa Bio). Expression values were calculated by a comparative Ct method, and U6 small nuclear RNA was used as miR-148b endogenous control, one primer is miRNA specific and the other is a universal primer. The glyceraldehyde-3-phosphate dehydrogenase (GAPDH) was used as WNT1 endogenous control. All qRT-PCR reactions were run in an Applied Biosystems 7500 Real-Time PCR System and performed in triplicate. The sequences of primers are shown in Table S1.

### Statistical Analysis

MiR-148b expression in HCC tissues and cells were compared by the unpaired Student's t test. The relationships between the expression of miR-148b and clinicopathologic factors were evaluated by χ^2^ test. HCC survival rates for miR-148b expression were estimated by using the Kaplan–Meier method, and the difference in survival curves were evaluated by log-rank test. The relationship between WNT1 and miR-148b was explored by Spearman's correlation. All statistical analyses were performed by SPSS13.0 software and all data were presented as means ± standard deviation (SD). Values were considered to be significantly different at p<0.05.

## Author Contributions

G.Z. and J.Z. conceived and designed the experiments. J.Z. performed the experiments. D.H. and C.W. analyzed the data. S.Y. and M.S. contributed reagents/materials/analytical tools. J.Z., D.H., G.Z. and D.H. wrote the manuscript. All authors reviewed the manuscript.

## Supplementary Material

Supplementary Information Suplementary Table and Figures

## Figures and Tables

**Table 1:** Relationship between miR-148b and clinical pathological characteristics in 40 patients with HCC

		miR-148b expression		
Parameters	Number of cases	High	Low	*P* value
Sex				
Male	27	13	14	0.736
Female	13	7	6	
Age (y)				
≥50	17	10	7	0.829
<50	23	10	13	
AFP (ng/ml)				
≥400	16	5	11	0.053
<400	24	15	9	
Tumor Size (cm)				
>5	25	8	17	0.003*
≤5	15	12	3	
histologic grade				
High/moderate	20	13	7	0.113
Poor	20	8	12	
TNM stage				
I	11	2	9	0.286
II~III	29	11	18	
HBV				
Positive	25	12	13	0.744
Negative	15	8	7	
Tumor number				
Single	22	15	7	0.011*
Two or more	18	5	13	
Metastasis				
Positive	17	3	14	<0.001*
Negative	23	17	6	

*Statistically significant (P<0.05). AFP: alpha-fetoprotein; TNM: tumor-node-metastasis staging system; HBV: hepatitis B virus.

## References

[b1] Parkin D. M., Bray F., Ferlay J. & Pisani P. Global cancer statistics, 2002. CA Cancer J Clin 55, 74–108 (2005). 15761078 10.3322/canjclin.55.2.74

[b2] Llovet J. M. & Bruix J. Molecular targeted therapies in hepatocellular carcinoma. Hepatology 48, 1312–1327 (2008). 18821591 10.1002/hep.22506 PMC2597642

[b3] Poon R. T. & Fan S. T. Hepatectomy for hepatocellular carcinoma: patient selection and postoperative outcome. Liver Transpl 10, S39–45 (2004). 14762838 10.1002/lt.20040

[b4] Chu R. *et al.* miRNAs affect the development of hepatocellular carcinoma via dysregulation of their biogenesis and expression. Cell Commun Signal 12, 45, doi: s12964-014-0045-y (2014). 25012758 10.1186/s12964-014-0045-y PMC4117189

[b5] Hoshida Y. *et al.* Integrative transcriptome analysis reveals common molecular subclasses of human hepatocellular carcinoma. Cancer Res 69, 7385–7392 (2009). 19723656 10.1158/0008-5472.CAN-09-1089 PMC3549578

[b6] Iwakawa H. O. & Tomari Y. Molecular insights into microRNA-mediated translational repression in plants. Mol Cell 52, 591–601 (2013). 24267452 10.1016/j.molcel.2013.10.033

[b7] Engels B. M. & Hutvagner G. Principles and effects of microRNA-mediated post-transcriptional gene regulation. Oncogene 25, 6163–6169 (2006). 17028595 10.1038/sj.onc.1209909

[b8] Bjorner S. *et al.* Epithelial and Stromal MicroRNA Signatures of Columnar Cell Hyperplasia Linking Let-7c to Precancerous and Cancerous Breast Cancer Cell Proliferation. PLoS One 9, e105099 (2014). 25122196 10.1371/journal.pone.0105099 PMC4133372

[b9] Gibb E. A., Brown C. J. & Lam W. L. The functional role of long non-coding RNA in human carcinomas. Mol Cancer 10, 38 (2011). 21489289 10.1186/1476-4598-10-38 PMC3098824

[b10] Budhu A. *et al.* Identification of metastasis-related microRNAs in hepatocellular carcinoma. Hepatology 47, 897–907 (2008). 18176954 10.1002/hep.22160

[b11] Hunt S., Jones A. V., Hinsley E. E., Whawell S. A. & Lambert D. W. MicroRNA-124 suppresses oral squamous cell carcinoma motility by targeting ITGB1. FEBS Lett 585, 187–192 (2011). 21112327 10.1016/j.febslet.2010.11.038

[b12] Iorio M. V. & Croce C. M. microRNA involvement in human cancer. Carcinogenesis 33, 1126–1133 (2012). 22491715 10.1093/carcin/bgs140 PMC3514864

[b13] Gramantieri L. *et al.* Cyclin G1 is a target of miR-122a, a microRNA frequently down-regulated in human hepatocellular carcinoma. Cancer Res 67, 6092–6099 (2007). 17616664 10.1158/0008-5472.CAN-06-4607

[b14] Yang Y. M. *et al.* Galpha gep oncogene deregulation of p53-responsive microRNAs promotes epithelial-mesenchymal transition of hepatocellular carcinoma. Oncogene 0, doi: 10.1038/onc.2014.218 onc2014218 (2014). 10.1038/onc.2014.218 25065598

[b15] Zhu K. *et al.* MiR-302c inhibits tumor growth of hepatocellular carcinoma by suppressing the endothelial-mesenchymal transition of endothelial cells. Sci Rep 4, 5524, doi: 10.1038/srep05524 srep05524 (2014). 25027009 10.1038/srep05524 PMC4100019

[b16] Song Y. *et al.* MicroRNA-148b suppresses cell growth by targeting cholecystokinin-2 receptor in colorectal cancer. Int J Cancer 131, 1042–1051 (2012). 22020560 10.1002/ijc.26485

[b17] Song Y. X. *et al.* MicroRNA-148b is frequently down-regulated in gastric cancer and acts as a tumor suppressor by inhibiting cell proliferation. Mol Cancer 10, 1 (2011). 21205300 10.1186/1476-4598-10-1 PMC3024301

[b18] Cimino D. *et al.* miR148b is a major coordinator of breast cancer progression in a relapse-associated microRNA signature by targeting ITGA5, ROCK1, PIK3CA, NRAS, and CSF1. FASEB J 27, 1223–1235 (2013). 23233531 10.1096/fj.12-214692

[b19] Bloomston M. *et al.* MicroRNA expression patterns to differentiate pancreatic adenocarcinoma from normal pancreas and chronic pancreatitis. JAMA 297, 1901–1908 (2007). 17473300 10.1001/jama.297.17.1901

[b20] Chang H. *et al.* Increased expression of miR-148b in ovarian carcinoma and its clinical significance. Mol Med Rep 5, 1277–1280 (2012). 22344713 10.3892/mmr.2012.794

[b21] Zhang Z., Zheng W. & Hai J. MicroRNA-148b expression is decreased in hepatocellular carcinoma and associated with prognosis. Med Oncol 31, 984 (2014). 24805877 10.1007/s12032-014-0984-6

[b22] Wieczorek M. *et al.* Silencing of Wnt-1 by siRNA induces apoptosis of MCF-7 human breast cancer cells. Cancer Biol Ther 7, 268–274 (2008). 18059186 10.4161/cbt.7.2.5300

[b23] Wang J. M. *et al.* Inhibition of Cancer Cell Migration and Invasion through Suppressing the Wnt1-mediating Signal Pathway by G-quadruplex Structure Stabilizers. J Biol Chem 289, 14612–14623 (2014). 24713700 10.1074/jbc.M114.548230 PMC4031517

[b24] Sasaya K., Sudo H., Maeda G., Kawashiri S. & Imai K. Concomitant loss of p120-catenin and beta-catenin membrane expression and oral carcinoma progression with E-cadherin reduction. PLoS One 8, e69777 (2013). 23936352 10.1371/journal.pone.0069777 PMC3735538

[b25] Xu X. *et al.* Aberrant Wnt1/beta-catenin expression is an independent poor prognostic marker of non-small cell lung cancer after surgery. J Thorac Oncol 6, 716–724 (2011). 21336184 10.1097/JTO.0b013e31820c5189

[b26] Ito T. *et al.* Survivin promotes cell proliferation in human hepatocellular carcinoma. Hepatology 31, 1080–1085 (2000). 10796883 10.1053/he.2000.6496

[b27] Masaki T. *et al.* Cyclins and cyclin-dependent kinases: comparative study of hepatocellular carcinoma versus cirrhosis. Hepatology 37, 534–543 (2003). 12601350 10.1053/jhep.2003.50112

[b28] Ramachandran I. *et al.* Wnt inhibitory factor 1 induces apoptosis and inhibits cervical cancer growth, invasion and angiogenesis in vivo. Oncogene 31, 2725–2737 (2012). 22002305 10.1038/onc.2011.455

[b29] Inagawa S. *et al.* Expression and prognostic roles of beta-catenin in hepatocellular carcinoma: correlation with tumor progression and postoperative survival. Clin Cancer Res 8, 450–456 (2002). 11839663

[b30] Endo K., Ueda T., Ueyama J., Ohta T. & Terada T. Immunoreactive E-cadherin, alpha-catenin, beta-catenin, and gamma-catenin proteins in hepatocellular carcinoma: relationships with tumor grade, clinicopathologic parameters, and patients' survival. Hum Pathol 31, 558–565 (2000). 10836294 10.1053/hp.2000.6683

[b31] Schotte D. *et al.* Identification of new microRNA genes and aberrant microRNA profiles in childhood acute lymphoblastic leukemia. Leukemia 23, 313–322 (2009). 18923441 10.1038/leu.2008.286

[b32] Lovat F., Valeri N. & Croce C. M. MicroRNAs in the pathogenesis of cancer. Semin Oncol 38, 724–733 (2011). 22082758 10.1053/j.seminoncol.2011.08.006

[b33] Dyrskjot L. *et al.* Genomic profiling of microRNAs in bladder cancer: miR-129 is associated with poor outcome and promotes cell death in vitro. Cancer Res 69, 4851–4860 (2009). 19487295 10.1158/0008-5472.CAN-08-4043

[b34] Slaby O. *et al.* Altered expression of miR-21, miR-31, miR-143 and miR-145 is related to clinicopathologic features of colorectal cancer. Oncology 72, 397–402 (2007). 18196926 10.1159/000113489

[b35] Zhao G. *et al.* miR-148b functions as a tumor suppressor in pancreatic cancer by targeting AMPKalpha1. Mol Cancer Ther 12, 83–93 (2013). 23171948 10.1158/1535-7163.MCT-12-0534-T

[b36] Cuk K. *et al.* Circulating microRNAs in plasma as early detection markers for breast cancer. Int J Cancer 132, 1602–1612 (2013). 22927033 10.1002/ijc.27799

[b37] Evan G. I. & Vousden K. H. Proliferation, cell cycle and apoptosis in cancer. Nature 411, 342–348 (2001). 11357141 10.1038/35077213

[b38] Mishra P. J. & Merlino G. MicroRNA reexpression as differentiation therapy in cancer. J Clin Invest 119, 2119–2123 (2009). 19620782 10.1172/JCI40107 PMC2719926

[b39] Breckenridge D. G. & Xue D. Regulation of mitochondrial membrane permeabilization by BCL-2 family proteins and caspases. Curr Opin Cell Biol 16, 647–52 (2004). 15530776 10.1016/j.ceb.2004.09.009

[b40] Benad P., Rauner M., Rachner T. D. & Hofbauer L. C. The anti-progestin RU-486 inhibits viability of MCF-7 breast cancer cells by suppressing WNT1. Cancer Lett 312, 101–108 (2011). 21899945 10.1016/j.canlet.2011.08.006

[b41] Lee T. H. *et al.* Enhanced nuclear factor-kappa B-associated Wnt-1 expression in hepatitis B- and C-related hepatocarcinogenesis: identification by functional proteomics. J Biomed Sci 13, 27–39 (2006). 16228287 10.1007/s11373-005-9030-1

[b42] Stanczak A. *et al.* Prognostic significance of Wnt-1, beta-catenin and E-cadherin expression in advanced colorectal carcinoma. Pathol Oncol Res 17, 955–963 (2011). 21678109 10.1007/s12253-011-9409-4 PMC3185231

[b43] Zhang J. G. *et al.* Sirtuin 1 facilitates chemoresistance of pancreatic cancer cells by regulating adaptive response to chemotherapy-induced stress. Cancer Sci 105, 445–454 (2014). 24484175 10.1111/cas.12364 PMC4317803

[b44] Zhang J. G. *et al.* Nicotinamide prohibits proliferation and enhances chemosensitivity of pancreatic cancer cells through deregulating SIRT1 and Ras/Akt pathways. Pancreatology 13, 140–146 (2013). 23561972 10.1016/j.pan.2013.01.001

[b45] Zhao G. *et al.* SIRT1 RNAi knockdown induces apoptosis and senescence, inhibits invasion and enhances chemosensitivity in pancreatic cancer cells. Gene Ther 18, 920–928 (2011). 21677689 10.1038/gt.2011.81

[b46] Zhang J. *et al.* microRNA-22, downregulated in hepatocellular carcinoma and correlated with prognosis, suppresses cell proliferation and tumourigenicity. Br J Cancer 103, 1215–1220 (2010). 20842113 10.1038/sj.bjc.6605895 PMC2967065

