# Corrigendum: MiR-148b suppresses cell proliferation and invasion in hepatocellular carcinoma by targeting WNT1/β-catenin pathway

**DOI:** 10.1038/srep46886

**Published:** 2017-08-24

**Authors:** Jun-gang Zhang, Ying Shi, De-fei Hong, Mengqi Song, Dongsheng Huang, Chun-you Wang, Gang Zhao

Scientific Reports 5: Article number: 8087; 10.1038/srep08087 published online: 01 28 2015; updated: 08 24 2017.

In the original version of this Article, an additional affiliation for Ying Shi was omitted. The correct affiliations are listed below:

Pancreatic Disease Institute, Union Hospital, Tongji Medical College, Huazhong University of Science and Technology, Wuhan, 430022, China.

Obstetrics and Gynecology, Zhejiang Provincial People’s Hospital, Hangzhou, 310014, China.

