# Supplementary material for: MiR-148b suppresses cell proliferation and invasion in hepatocellular carcinoma by targeting WNT1/β-catenin pathway
Source: Sci Rep. 2015 Jan 28;5:8087. doi: 10.1038/srep08087 (PMC4310092; doi:10.1038/srep08087)
Supplement: Supplementary Information — Suplementary Table and Figures [file srep08087-s1.pdf]

MiR-148b suppresses cell proliferation and invasion in hepatocellular carcinoma by targeting Wnt1/β-catenin pathway

Jun-gang Zhang[1, 2, #], Ying Shi[3, #], De-fei Hong[1], Mengqi Song[4], Dongsheng Huang[1, *] Chun-you Wang[1, *], Gang Zhao[1,*]

[1] Pancreatic Disease Institute, Union Hospital, Tongji Medical College, Huazhong University of Science and Technology, Wuhan, 430022, China.

[2] Hepatobiliary and Pancreatic Surgery, Zhejiang Provincial People's Hospital, Hangzhou, 310014, China.

[3] Obstetrics and Gynecology, Zhejiang Provincial People's Hospital, Hangzhou, 310014 , China.

[4] Hepatobiliary Surgery, Union Hospital, Tongji Medical College, Huazhong University of Science and Technology, Wuhan, 430022, China.

[#] These authors contributed equally to this work

* Corresponding author: Prof. Gang Zhao[1], Prof. Chun-you Wang[1], Prof. Dongsheng Huang[2]

[1] Pancreatic Disease Institute, Union Hospital, Jiefang Avenue 1277, Wuhan City, Hubei Province 430022, China

[2] Hepatobiliary and Pancreatic Surgery, Zhejiang Provincial People's Hospital, Shangtang Road 158, Hangzhou, Zhejiang Province 310014, China.

E-mail: gangzhao@aliyun.com, yishengdongsheng1@yeah.net.

Table S1 Sequences of qRT-PCR primers

Primer	Primer Sequence
WNT1-F[a]	5’- CTCATGAACCTTCACAACAACGA-3’
WNT1-R[b]	5’- ATCCCGTGGCACTTGCA-3’
GAPDH-F[a]	5’-GAAGGTGAAGGTCGGAGTC-3’
GAPDH-R[b]	5’-GAAGATGGTGATGGGATT-3’
miR-148b-F[a]	5’-AAGTTCTGTTATACACTCAGGC-3’
U6-F[a]	5’-CTCGCTTCGGCAGCACA-3’

[a] Forward primer

[b] Reverse primer

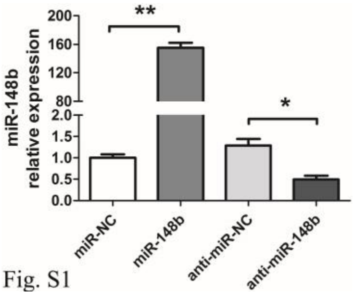

Fig. S1

Figure S1. Modifications of miR-148b levels in HCC cells. The expression levels of miR-148b were tested by qRT-PCR in HepG2 cells transfected with miR-148b mimics (miR-148b), anti-miR-148b and their respective NCs (50nM) for 48h.

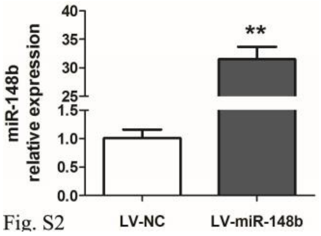

Fig. S2

Figure S2. The expression levels of miR-148b in excised tumors were analyzed by qRT-PCR, and the expression was normalized to the endogenous control (U6 RNA). Data were shown as the Mean±SD of 10 mice. *P<0.05; **P<0.01 compared with control.

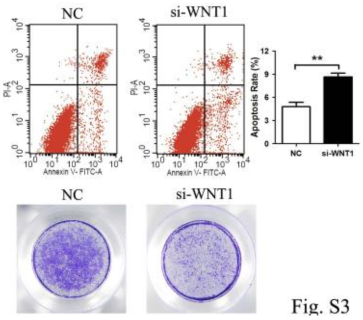

Fig. S3

Figure S3. Downregulation of WNT1 increased apoptosis and inhibited invasion of HCC cells. (A) HepG2 cells were transfected with si-WNT1 or NC. After 48 h, the percentage of apoptotic cells was determined by flow cytometry of Annexin V/PI staining cells. (B) HepG2 cells were treated as above, and the cell invasive ability was measured by matrigel invasion assays. Quantification was performed by counting the stained cells invading to the lower chamber under the light microscopy. Data shown were representative of three independent experiments. ** P<0.01 compared with control.
